# Exploring the Role of Bipolar Thermal Radiofrequency in Complex Multi-pattern Post-stroke Spasticity: Clinical Insights From a Case Report With Combined Botulinum Toxin Type A Therapy

**DOI:** 10.7759/cureus.97613

**Published:** 2025-11-23

**Authors:** Carlos Cordero-García, Marta Lopera Martínez, Blanca Cabaleiro Burguillos, María del Mar Saenz de Tejada Sánchez

**Affiliations:** 1 Physical Medicine and Rehabilitation, Juan Ramón Jiménez University Hospital, Huelva, ESP

**Keywords:** botulinum toxin, high-dose, incobotulinumtoxina, neurolysis, radiofrequency, spasticity, stroke

## Abstract

Post-stroke spasticity significantly impairs quality of life by hindering functional abilities and increasing dependency. Common treatments include botulinum toxin type A (BoNTA) injections. BoNTA is effective for spasticity management, but some situations may require alternative approaches. This case report explores the role and efficacy of bipolar thermal radiofrequency in complex multi-pattern post-stroke spasticity.

A 44-year-old man experienced increased left-sided spasticity five years post-stroke, despite initial success with BoNTA. The patient exhibited left hemiparesis with severe spasticity in the left limbs (Modified Ashworth Scale (MAS) score 2 to 4 in several muscle groups) and was treated with high doses of incobotulinumtoxinA. Combined treatment with bipolar thermal radiofrequency was implemented to redistribute the dose among other muscles, achieving a more comprehensive approach for better outcomes. It resulted in immediate spasticity reduction in elbow flexors (MAS 2 to 0). Four weeks post-treatment, the patient reported pain disappearance (visual analogue scale (VAS) 0) and reduced spasticity (MAS 0-3 in several muscle groups).

In conclusion, thermal radiofrequency enhances outcomes in post-stroke spasticity, potentially improving functionality and quality of life. As one of the first cases reported, our findings highlight its feasibility and potential benefit. Further research is needed to explore broader applications.

## Introduction

A stroke, or cerebrovascular accident, occurs when blood flow to the brain is disrupted due to either a blockage (ischemic stroke) or a vessel rupture (hemorrhagic stroke). This leads to an acute episode of focal dysfunction of the brain, also encompassing subarachnoid hemorrhage [1]. Spasticity, often developing after initial post-stroke weakness, is a motor disorder marked by velocity-dependent muscle hypertonia from stretch reflex hyperexcitability [2]. Although it might be expected that stroke type could influence the development of post-stroke spasticity, current evidence shows no conclusive correlation among ischemic, hemorrhagic, or subarachnoid strokes. Instead, factors such as lesion location and lesion severity appear to play a more decisive role [1]. It progressively worsens, contributing to contractures, abnormal joint postures, and impaired mobility, complicating daily tasks. In spastic hemiparesis, involuntary muscle activation across multiple joints leads to common patterns, such as elbow flexion involving the biceps, brachialis, and brachioradialis. These synergistic movements hinder functional tasks like reaching, with greater impairment as spasticity increases [2].

Spasticity affects 27-36% of stroke survivors within the first year [1], negatively impacting quality of life by limiting functional abilities, restricting basic activities of daily living (ADLs), and increasing dependency [3]. Pain is also common and often severe, complicating spasticity treatment as it may hinder rehabilitation; therefore, its management is essential for stroke patients [3]. Treatment should be individualized with a multidisciplinary approach, aiming to improve function, ease self-care, reduce pain, prevent complications, and enhance quality of life [3]. Key considerations include the severity and distribution of spasticity, the stage of stroke recovery, and the type of intervention selected according to the needs of the patient [2].

Spasticity management has significantly evolved, now encompassing treatment options such as physical therapy, pharmacological treatment (botulinum toxin type A (BoNTA), baclofen), neurolysis, and surgical treatment [4]. Intramuscular BoNTA injections effectively improve upper and lower limb mobility and enhance patient autonomy. The safe and effective use of higher-than-labeled incobotulinumtoxinA doses has been reported [5]; however, in some cases, radiofrequency could be a potential combined approach to redistribute the dose among other muscles, achieving a more comprehensive treatment in patients who are already receiving a high toxin dose [6].

Percutaneous neurolysis techniques include chemical agents (alcohol, phenol) and thermal methods like radiofrequency and cryoneurolysis [7]. Radiofrequency therapy is typically classified into three main types: thermal, pulsed, and cold. Thermal radiofrequency delivers continuous high-frequency current to generate heat, producing a thermal lesion that ablates nerve tissue. In contrast, pulsed radiofrequency applies short bursts of current at lower temperatures (typically ≤42°C), modulating nerve activity without destroying tissue, while cold radiofrequency uses internally cooled probes to allow controlled, larger lesion sizes at lower tissue temperatures. Radiofrequency has demonstrated efficacy and safety under proper conditions for chronic pain management [8]. However, its application for spasticity remains limited, with most studies focusing on axial targets such as dorsal or lumbar roots, ganglia, or rhizotomy [9]. Despite being one of its potential applications, motor nerve ablation is rarely employed for peripheral nerves, and evidence is still scarce [9,10]. In selected cases, successful sensory nerve ablation may support its inclusion in multimodal strategies for motor nerve spasticity treatment. Results showed improvements in spasticity, pain, function, and quality of life with minimal adverse effects [9]. While not a first-line option, peripheral radiofrequency ablation may complement BoNTA therapy in selected patients [10].

Among the BoNTA formulations available, incobotulinumtoxinA (Xeomin®, Merz Therapeutics GmbH, Frankfurt am Main, Germany) is free of complexing proteins, leading to a lower antigenicity compared to onabotulinumtoxinA and abobotulinumtoxinA. These complexing proteins are necessary for the passage of the acidic milieu of the stomach and penetration through the gastrointestinal wall, but they are not needed for the therapeutic muscle-relaxing action of the toxin. In contrast, these complex proteins may stimulate the immune system, acting as adjuvant substances, and they might hinder rather than enhance the effectiveness of botulinum toxin [11]. Due to the proven tolerability, lack of secondary nonresponse in pivotal clinical trials, and high purity, incobotulinumtoxinA is a suitable BoNTA formulation for a high-dose treatment [5]. The TOWER study confirmed the safety and efficacy of incobotulinumtoxinA doses up to 800U [12]. When clinically required, total doses up to 1500U have been reported in specialized centers [6].

To explore alternatives for managing multi-pattern spasticity unresponsive to maximum BoNTA doses, we investigated the role and efficacy of thermal radiofrequency. To the best of our knowledge, this study represents one of the pioneering investigations applying radiofrequency to peripheral motor nerve branches in patients suffering from severe chronic post-stroke spasticity, in combination with BoNTA therapy.

## Case presentation

A 44-year-old man experienced a right pontine and cerebellar ischemic stroke in July 2016. Following physical and occupational therapy, the patient presented left-sided hemiparesis and multi-pattern spasticity, which was initially managed with incobotulinumtoxinA, obtaining a good response.

Five years later, the patient exhibited increased spasticity, particularly pronounced in the left upper limb, requiring treatment for a greater number of spastic patterns. Clinical examination revealed left hemiparesis and severe equinovarus deformity of the foot. The patient required assistance for standing and was unable to perform functional walking. Spasticity was observed in the left hemibody with a Modified Ashworth Scale (MAS) [13,14] score of 2 in the hamstrings, MAS 3 in the extensor hallucis longus, MAS 4 in the posterior tibial muscle, MAS 2 in the toe flexors, and MAS 2 in the elbow flexors.

**Figure 1:**
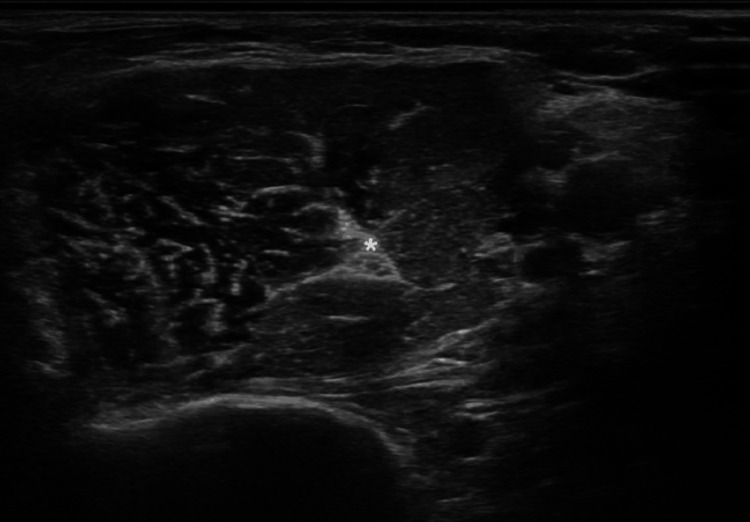
Transverse ultrasound section showing the musculocutaneous nerve (*) in the thickness of the coracobrachialis muscle.

**Figure 2:**
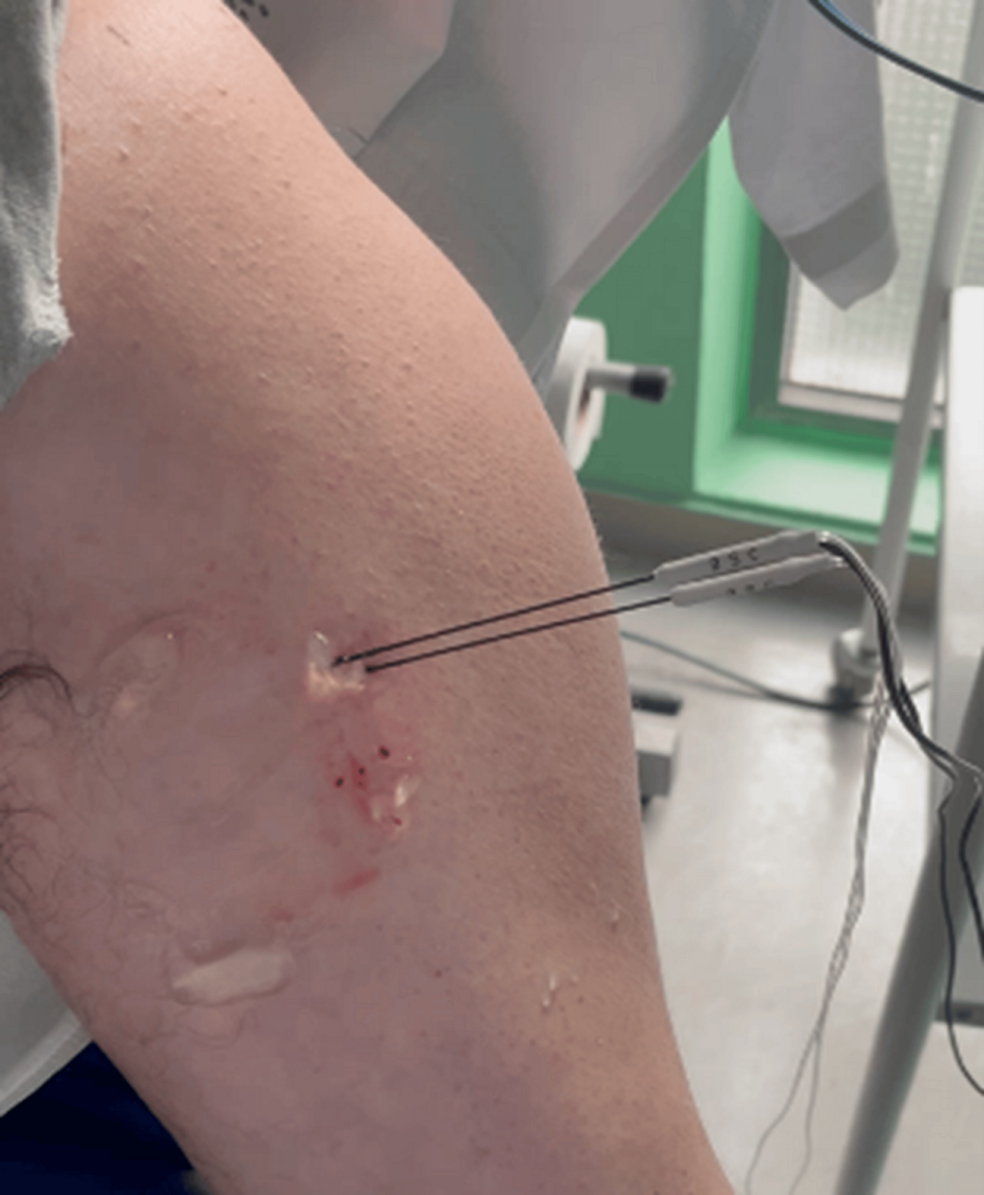
Thermal bipolar radiofrequency ablation on the musculocutaneous nerve.

The patient was treated with repeated cycles of incobotulinumtoxinA every 12 weeks, with the following doses and locations: 180 units in the gastrocnemius (90U each), 90U in the soleus, 100U in the tibialis posterior muscle, 80U in the semimembranosus, 80U in the semitendinosus, 80U in the extensor hallucis longus, 120U in the biceps brachii, and 50U in the brachialis (total body dose 780U). Although partial improvement was observed, the results did not meet the treatment goals in the latest infiltration cycles. Given that the patient was already being treated with high doses of BoNTA, a new therapeutic approach was considered for the multimodal management of spasticity. Based on this, it was decided to address the spastic flexion pattern of the elbow in a differentiated manner. Initially, an ultrasound-guided block of the left musculocutaneous nerve was performed using 8 cc of 1.5% mepivacaine. Immediately after the block, the patient exhibited a complete resolution of elbow flexor spasticity (MAS 2 to MAS 0). Consequently, conventional bipolar radiofrequency ablation was proposed on the musculocutaneous nerve at 83°C for 120 seconds, under ultrasound guidance, following correct sensory-motor stimulation and aseptic conditions. A transverse ultrasound section showing the musculocutaneous nerve is shown in Figure 1. The procedure utilized a Boston Scientific G4TM radiofrequency generator (Boston Scientific Corporation, Marlborough, MA) and a 10 cm x 10 mm x 22 ga (0.7 mm) Boston Scientific injection electrode cannula (Boston Scientific Corporation, Marlborough, MA) (Figure 2).

IncobotulinumtoxinA doses were redistributed as follows: 200U in the gastrocnemius (100U each), 100U in the tibialis posterior muscle, 80U in the extensor hallucis longus, 80U in the common toe flexor, 60U in the semimembranosus, and 60U in the semitendinosus. Four weeks after the combined application of radiofrequency and BoNTA treatment, the patient reported a disappearance of pain (visual analogue scale (VAS) 0) and a reduction in spasticity (MAS 0 in elbow flexion, MAS 1 in toe flexors, MAS 1 in knee flexors, MAS 3 in plantar flexors and tibialis posterior muscle, and MAS 1 in the extensor hallucis longus). No adverse or safety effects were recorded following the implementation of the technique. Written informed consent was obtained from the patient for publication of this case report and the accompanying images.

## Discussion

Post-stroke spasticity causes a significant decline in patients' quality of life; therefore, proper management is essential. Conventional treatments include physical therapy, oral medication, and BoNTA. However, managing multipattern spasticity can be challenging, and adjunctive treatments should be considered for patients who do not respond positively to these therapies. To the best of our knowledge, no cases have been found where radiofrequency is used as an adjuvant treatment that allows BoNTA to redistribute dosing in the treatment of post-stroke spasticity. In this case report, BoNTA treatment facilitated care for years, but in the last cycles, spasticity progression and subsequent reductions in the treatment efficacy and the need for higher doses led us to propose radiofrequency as an adjuvant.

Thermal radiofrequency is a medical procedure that uses heat generated by radiofrequency energy to target and treat specific nerve tissues. This technique is primarily used to manage chronic pain by disrupting pain signal transmission. The heat is applied through a needle electrode, which is precisely positioned near the nerve, aiming to create a small, controlled lesion and reduce its ability to transmit pain or motor signals without causing significant damage to the surrounding tissues [8].

Since thermal radiofrequency has been proven to be a successful treatment for sensory nerve pain, we propose its use in motor nerves within a multimodal approach for spasticity. However, as stated before, while the available literature for the use of radiofrequency in pain is wide, its use in spasticity is scarce [9,15]. Research on neurolysis for post-stroke spasticity has mainly focused on chemical methods, particularly phenol and alcohol. Studies show that chemical neurolysis can effectively reduce spasticity and improve functional outcomes. Phenol neurolysis has been widely used in reducing spasticity, preventing contractures, and improving gait [16]. Comparative studies indicate that both phenol and alcohol are effective in reducing spasticity, with alcohol offering slightly longer-lasting relief [17]. Alcohol neurolysis is noted for its safety and therapeutic benefits, especially in managing spasticity that impedes care and causes pain [17]. Research also shows that phenol neurolysis is effective for focal spasticity in the distal upper extremity, with safety ensured through precise localization techniques like ultrasound and electrical stimulation [18]. Additionally, a novel approach using high-frequency ultrasound imaging for selective peripheral neurolysis has been introduced to reduce side effects by targeting motor branches while sparing cutaneous nerves. Overall, these methods provide valuable insights for the management of post-stroke spasticity with a focus on efficacy and safety. While innovative, this approach still relies on chemical agents, reaffirming the dominance of chemical neurolysis in the current research landscape [19]. Therefore, although chemical neurolysis with phenol could be a viable option, it typically results in larger tissue lesions. In contrast, thermal radiofrequency offers similar therapeutic benefits while minimizing damage to surrounding tissues. Before thermal radiofrequency, nerve blocks with local anesthetics are administered, enabling the prediction of treatment outcomes. Recent data on thermal radiofrequency applied to the musculocutaneous nerve reported mild dysesthesia in two of 12 patients (16%), localized to the lateral forearm and resolving completely within two to four weeks, supporting the conclusion that adverse effects are infrequent, mild, and self-limiting [9].

Radiofrequency ablation could allow for redistribution of the frequency and doses of the toxin injections. This combined approach could optimize patient outcomes by leveraging the strengths of both therapies. Thipayawat et al. [20] have suggested that radiofrequency ablation targeting motor branches of nerves may provide an additional treatment to BoNTA injections for spasticity. This suggests a potential complementary role for both techniques, where radiofrequency could be used to create a more permanent reduction in nerve activity, and BoNTA could be employed for more targeted, short-term modulation of muscle tone. Closely related, some studies have involved the treatment of spasticity in patients with spinal cord injury. One notable case by Pascoal et al. [15] examines the use of ultrasound-guided percutaneous radiofrequency thermal neuroablation to treat adductor and rectus femoris spasticity in a 60-year-old female patient with complete spastic paraplegia following a spinal stroke. The patient's spasticity was significantly interfering with ADLs, particularly with intermittent catheterization and transfers, and BoNTA injections failed to provide long-term relief. In this case, radiofrequency ablation was performed on the anterior and posterior branches of the obturator nerve and motor branches to the rectus femoris of the femoral nerve. One year after the radiofrequency treatment, the patient showed a considerable reduction in spasticity​​. The successful use of radiofrequency ablation in spinal cord injury patients suggests a potential application for post-stroke spasticity. Indeed, recent research by Sergio Otero-Villaverde et al. [9] explored the application of thermal radiofrequency to the musculocutaneous nerve for treating severe elbow flexor spasticity. The patients were refractory to conventional physical therapy and BoNTA, without reaching the goals set in regard to the elbow flexor pattern. Results showed significant improvements in spasticity, pain, and overall functioning, with high satisfaction reported by both patients and physicians. Treatment goals were successfully achieved, and minimal side effects were observed. This study highlights thermal radiofrequency as a safe and effective technique for managing post-stroke spasticity. However, its use in this setting has remained limited, likely due to the technical expertise required for precise nerve targeting, the absence of standardized procedural guidelines, and the lack of large-scale studies providing consistent clinical evidence on its efficacy and safety.

## Conclusions

Radiofrequency ablation could potentially enhance the effects of BoNTA by targeting specific nerves and reducing the overall spasticity more effectively. This case is the first described with a bipolar configuration, an approach that can improve the distribution of the required doses of the toxin, leading to more effective and longer-lasting relief and improving patient outcomes. However, further studies with scientifically significant results are needed to confirm these findings and establish the clinical relevance of this combined approach.
